# Case Report: Upadacitinib for SAPHO syndrome with biologics-induced paradoxical manifestation and Hyperimmunoglobulinemia E

**DOI:** 10.3389/fimmu.2026.1728245

**Published:** 2026-02-05

**Authors:** Yuxi Liu, Di Jin, Wen Li, Shiqi Zhang, Chen Li, Xin Li

**Affiliations:** 1 Department of Critical Rheumatology and Immunology, Clinical Immunology Centre, Tianjin Medical University General Hospital, Tianjin, China; 2 Department of Rheumatology, Weifang People’s Hospital, Weifang, Shandong, China; 3 School of traditional Chinese medicine, Beijing University of Chinese Medicine, Beijing, China; 4 Department of Dermatology, Tianjin Institute of Integrative Dermatology, Tianjin Academy of Traditional Chinese Medicine Affiliated Hospital, Tianjin, China

**Keywords:** Hyperimmunoglobulinemia E, JAK inhibitors, paradoxical manifestation, SAPHO syndrome, upadacitinib

## Abstract

**Background:**

SAPHO (synovitis, acne, pustulosis, hyperostosis, and osteitis) syndrome is a rare autoinflammatory disease. Paradoxical reactions and immune deviation following biologic therapy are occasionally observed in clinical practice; however, to our knowledge, no previous cases of paradoxical skin rash accompanied by Hyperimmunoglobulinemia E have been reported.

**Case presentation:**

A 35-year-old male with SAPHO syndrome experienced significant exacerbation of palmoplantar pustulosis and a sharp increase in immunoglobulin E(IgE) levels following treatment with secukinumab and infliximab. After one month of upadacitinib therapy, the patient showed marked improvement in cutaneous lesions and bone pain, along with significant normalization of inflammatory markers and a pronounced reduction in IgE levels.

**Conclusion:**

This case suggests that upadacitinib may be a valuable therapeutic option for patients with refractory SAPHO syndrome, particularly those presenting with paradoxical reactions or hypersensitivity. The unique mechanism of action of Janus kinase (JAK) inhibitors may offer superior efficacy in such cases, though further clinical validation and mechanistic studies are warranted.

## Introduction

SAPHO(synovitis, acne, pustulosis, hyperostosis, and osteitis) syndrome is a rare autoinflammatory disorder characterized by synchronous skin-bone-joint inflammation, presenting clinically with synovitis, acne, pustulosis, hyperostosis, and osteitis (1). Its pathogenesis remains unclear, potentially involving individual genetic differences, immune status, microbial factors, and environmental influences (2).SAPHO syndrome exhibits clinical heterogeneity, rendering diagnosis challenging. Commonly employed therapeutic approaches include antibacterial agents, non-steroidal anti-inflammatory drugs (NSAIDs), corticosteroids, bisphosphonates, and conventional disease-modifying antirheumatic drugs (cDMARDs) such as methotrexate. In recent years, biologic agents such as tumor necrosis factor(TNF)-α inhibitors and interleukin-17/-interleukin23(IL-17/IL-23) inhibitors have become increasingly important therapeutic options (3). However, reports indicate paradoxical manifestations in some patients, though no cases of allergic reactions or Hyperimmunoglobulinemia E have been observed to date. Upadacitinib is a highly selective Janus kinase(JAK)1 inhibitor approved for treating rheumatoid arthritis, psoriatic arthritis, atopic dermatitis, and other conditions (4), demonstrating unique advantages in refractory inflammatory diseases (5). This paper presents the first reported case of upadacitinib treating SAPHO syndrome complicated by biologic-induced paradoxical rash and abnormally elevated immunoglobulin E(IgE) levels. It aims to explore the clinical efficacy and therapeutic targeting of upadacitinib in this specific status, offering a potential treatment option for SAPHO patients who have failed biologic therapy.

## Case presentation

**Figure 1:**
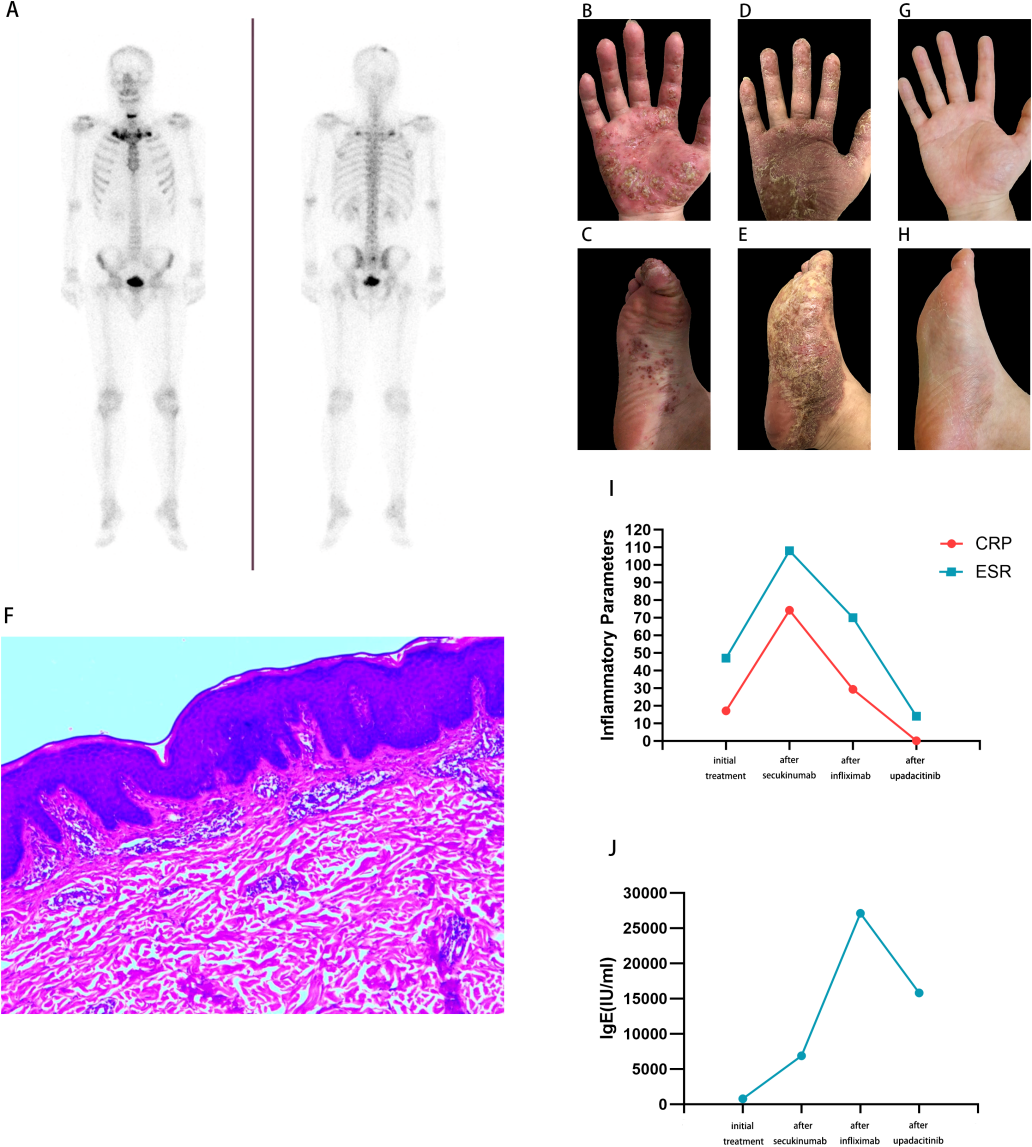
Patient’s imaging and pathological examination, skin manifestations and changes in indicators. **(A)** Whole-body bone scintigraphy revealed abnormal tracer accumulation in bilateral costal regions, the sternoclavicular joint areas, the sacrum, and bilateral sacroiliac joints, presenting the classic “bull’s head sign”; **(B)** Exacerbation of hands pustular psoriasis following treatment with secukinumab; **(C)** Exacerbation of pustular psoriasis on the feet following treatment with secukinumab; **(D)** After treatment with infliximab, the hands rash worsened again, accompanied by itching, crusting, and desquamation; **(E)** After treatment with infliximab, the feet rash worsened again, accompanied by itching, crusting, and desquamation; **(F)** Skin biopsy revealed hyperkeratosis of the epidermis, mild hyperplasia of the granular and spinous layers, Elongation of the rete ridges, and perivascular lymphocytic infiltration in the dermis; **(G)** The hands rash has markedly resolved following treatment with upadacitinib; **(H)** The feet rash has markedly resolved following treatment with upadacitinib; **(I)** Changes in inflammatory parameters levels during treatment; **(J)** Changes in IgE levels during treatment.

Initial presentation and diagnosis(April 2022): The patient is a 35-year-old male who presented three years ago with anterior chest and lower back pain, accompanied by herpetic lesions with ulceration on both hands and feet. Physical examination revealed tenderness over both costal margins and sternoclavicular joints; tenderness between the spinous processes of L5 and S1 vertebrae. Papular vesicles were observed on the palms of both hands and soles of both feet, which ruptured to discharge yellowish fluid, accompanied by crusting and desquamation, with visible erythematous ecchymoses. Whole-body bone scintigraphy revealed abnormal tracer concentration in bilateral articulations sternocostales regions, the sternoclavicular joint areas, the sacrum, and bilateral sacroiliac joints, presenting the classic “bull’s head sign” (**Figure 1A**). Laboratory investigations revealed an erythrocyte sedimentation rate (ESR) of 47 mm/h, C-reactive protein (CRP) at 17.2 mg/L, and IgE 778.00 IU/mL. Other laboratory results are detailed in **Table 1**. Diagnosis of SAPHO syndrome was confirmed.

**Table 1:** Laboratory results at initial treatment.

Inspection item	Result	Reference range
White blood cells (10^9/L)	8.78	3.5~9.5
Red blood cells (10^9/L)	4.19	4.3~5.8
Hemoglobin (g/L)	126	130~175
Platelets (10^9/L)	340	125~350
CRP (mg/L)	17.2	0~5
ESR (mm/h)	47	0~15
Total protein (g/L)	73	65~85
Albumin (g/L)	34	40~55
AST (IU/l)	43	15~40
ALT (IU/l)	19	9~50
CREA (umol/L)	42	57~111
IgG (mg/dl)	1240	751~1560
IgE (IU/ml)	778	<165
C3 (mg/dl)	152	79~152
C4 (mg/dl)	42.5	16~38
IL-6 (pg/ml)	23.86	0.00~5.30
ANA	(−)	
Anti-dsDNA Ab (IU/ml)	(−)	
Anti-sm Ab (U/ml)	(−)	
Anti-SSA Ab (U/ml)	(−)	
Anti-RNP Ab (U/ml)	(−)	
Anti-platelet Ab	(−)	
Protein	(−)	
Urine glucose	(−)	
Uric blood	(−)	
Urinary cast	(−)	
24h protein(mg/24h)	264	

WBC, White blood cells; RBC, Red blood cells; CRP, C-reactive protein; ESR, erythrocyte sedimentation rate; AST, aspartate aminotransferase; ALT, alanine aminotransferase; CREA, creatine; IgG, immunoglobulin G; IgE, immunoglobulin E; RF, rheumatoid factor; ANA, antinuclear antibody; Ab, antibody; Anti-sm, anti-smith; Anti-RNP, anti ribonucleoprotein; Anti-SSA, anti Sjogren syndrome antigen; IL, interleukin; TNF, tumor necrosis factor.

First-line therapy and partial response: Initial treatment was commenced with loxoprofen sodium tablets (60 mg po twice a day), methotrexate (10 mg po once a week), and alendronate sodium tablets (70 mg po once a week). The patient’s symptoms improved.

Paradoxical reaction under IL-17 inhibitor (September 2022): After five months of treatment, the patient experienced worsening shoulder and back pain, with herpes lesions spreading from the plantar surface to the dorsum of the feet. Treatment was continued with secukinumab (300 mg S.C once a week) for one month. Follow-up blood tests revealed an ESR 108mm/h, CRP 74.3 mg/L, IgE 6880.00 IU/mL. The patient reported worsening of shoulder-back and lumbar-gluteal pain, extending to both arms. Pustular lesions appeared on the palms and soles, worsening compared to previous episodes (**Figures 1B, C**). This was considered a paradoxical reaction to IL-17 inhibitors. Thereafter, the patient intermittently and irregularly received secukinumab until March 2025, achieving clinical symptom remission, though serum IgE levels remained persistently elevated.

Worsening and hypersensitivity reaction under TNF-α inhibition (April 2025): Due to persistently elevated IgE levels, therapy was switched to infliximab (100mg IV 3d). Following the third infusion, the patient developed pain in the anterior chest and buttocks, with a recurrence and worsening of the generalized rash, accompanied by pruritus, crusting, and desquamation (**Figures 1D, E**). Follow-up blood tests revealed an ESR 70 mm/h, CRP 29.3 mg/L, IgE 27,100.00 IU/mL. Skin biopsy revealed hyperkeratosis of the epidermis, mild hyperplasia of the granular and spinous layers, Elongation of the rete ridges, and perivascular lymphocytic infiltration in the dermis (**Figure 1F**).

Diagnostic reassessment: The patient was considered to have developed a paradoxical rash and allergic reaction following the administration of biologic agents. Infliximab was discontinued. Following one week of symptomatic treatment with anti-allergy medication, the rash has shown slight improvement.

Initiation of JAK inhibitor and therapeutic response (May 2025): Treatment with upadacitinib (15mg po once a day) was then initiated. One month later, follow-up revealed an ESR 14 mm/h, CRP <0.2 mg/L, and IgE 15,800 IU/mL. The patient’s rash had markedly subsided, and bone pain had resolved (**Figure 1G, H**). Changes in inflammatory parameters and IgE levels during treatment are shown in **Figures 1I and J**.

**Figure 2:**
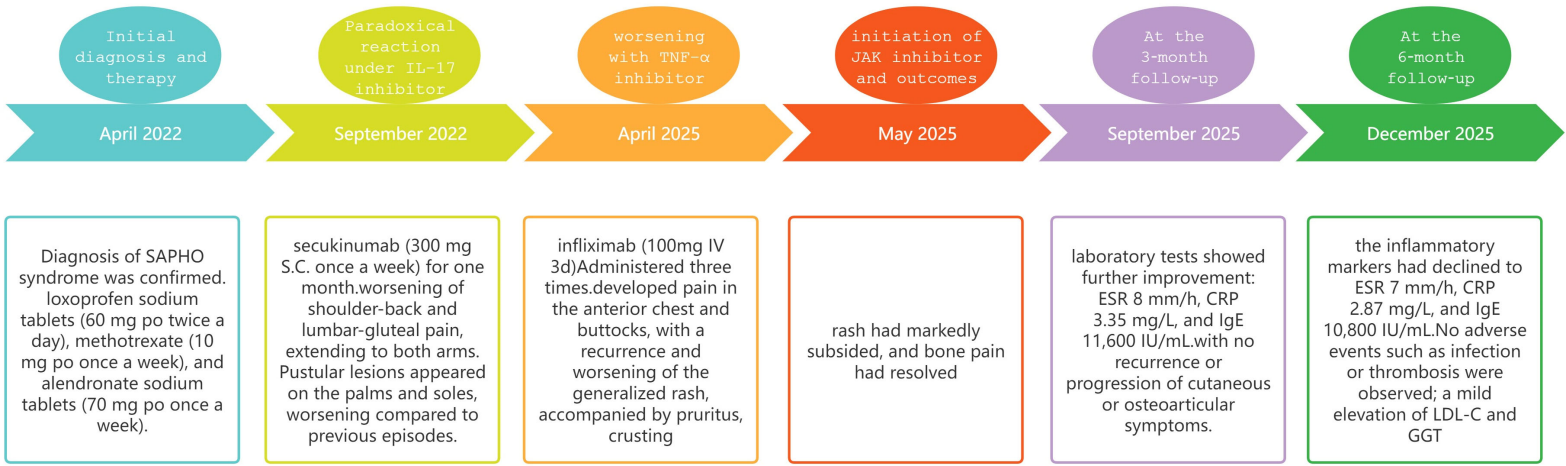
The timeline chart illustrates the sequence of treatment and the occurrence and progression of paradoxical manifestations, providing a clearer overview of the clinical course. ESR: erythrocyte sedimentation rate, CRP: C-reactive protein, IgE: immunoglobulin E, LDL−C: low-density lipoprotein cholesterol, GGT: γ-glutamyl transferase.

Follow-up and long-term response: While drafting and revising this article, the patient continued upadacitinib therapy with sustained clinical stability. At the 3−month follow−up (September 2025), laboratory tests showed further improvement: ESR 8mm/h, CRP 3.35mg/L, and IgE 11,600IU/mL. By the 6−month follow−up (December 2025), the inflammatory markers had declined to ESR 7mm/h, CRP 2.87mg/L, and IgE 10,800IU/mL, with no recurrence or progression of cutaneous or osteoarticular symptoms. No adverse events such as infection or thrombosis were observed; however, a mild elevation of low-density lipoprotein cholesterol (LDL−C) and γ-glutamyl transferase (GGT) was noted at the 6−month assessment. To provide a clearer overview of the clinical course, illustrating the sequence of treatment and the occurrence and progression of paradoxical manifestations, a timeline diagram is presented in **Figure 2**.

The patient reported feeling markedly better after switching to upadacitinib, with resolution of the skin rash and relief of bone pain. The improvement in symptoms led to a substantial enhancement in his overall quality of life and sleep.

## Discussion

Currently, there is no universally accepted treatment protocol for SAPHO syndrome. Although biologics such as TNF-α inhibitors (e.g., infliximab) and IL-17/23 inhibitors (e.g., secukinumab) have demonstrated therapeutic potential, their efficacy varies considerably between individuals. In clinical practice, immune drift, treatment failure, or paradoxical effects are frequently observed. A considerable body of literature has documented these findings, with relevant summaries presented in **Table 2**.

**Table 2:** Literature summary of biologics-induced paradoxical manifestations in SAPHO syndrome.

Pt	Sex	Age(yrs)	Biological agent	Duration of therapy(weeks)	Paradoxical skin lesions	Inflammatory markers	Management	Time to remission(weeks)
1~2 (20)	M	43/47	Infliximab	6/24	Relapse or exacerbation of PPP	reduce	suspend, steroids	/
3 (21)	F	7	Infliximab	24	suspected fungal skin infection	/	suspend	6
4 (22)	F	48	Infliximab	14	psoriasiform skin lesions	/	/	8
5 (23)	F	58	Etanercept	4	new psoriasiform lesions and exacerbations of PPP	reduce	TwHF	4
6~12 (24)	F/M	means 46.7	Infliximab or Etanercept	3~10	new psoriasiform lesions; alopecia; PPP aggravate,	/	suspend/vitamin D analogs/DMARDs	8~16
13 (25)	M	38	Adalimumab	8	facial infection, new pustules on the trunk	/	Ustekinumab	12
14 (26)	F	31	Adalimumab	4	psoriasiform rash,PPPASI6.6	/	Secukinumab	16
15 (27)	F	63	Secukinumab	3	pustule,erythema multiforme,PASI 18.3	/	Risankizumab	16
16~18 (28)	F	means 47.7	Secukinumab	12~16	New onset or exacerbation of pustular skin lesions	reduce	Tofacitinib	4
19 (13)	F	46	Secukinumab	12	paradoxical psoriasis	/	suspend	/
20 (29)	F	24	Secukinumab	12	generalized pustular skin rash,drug hypersensitivity reaction	/	apremilast	4
21 (30)	F	31	Secukinumab	2	exacerbation of PPP,PPPASI 31.2	/	Tofacitinib	2
22 (13)	F	37	Ustekinumab	24	Paradoxical psoriasis	/	suspend	/
23 (31)	M	78	Tocilizumab	3	subcutaneous abscess on the anterior chest,aseptic abscess	reduce	Infliximab	/
24 (32)	F	29	Tocilizumab	4	new-onset scattered pustular rashes	reduce	Bisphosphonate and minocycline	/

PPP, palmoplantar pustulosis; PASI, PPPASI, palmoplantar pustulosis area and severity index; TwHF, tripterygium wilfordii hook; DMARDs, disease-modifying antirheumatic drugs.

The exacerbation of palmoplantar pustulosis (PPP) following biologic therapy remains incompletely understood, though it is hypothesized to represent a severe allergic reaction associated with T helper 2 (Th2)-type inflammation, which is characterized by elevated eosinophil counts and/or high serum IgE levels (6). The pathogenesis of PPP is centrally driven by Th2 immune responses, operating independently of the IL-23/Th17 axis characteristic of classical psoriasis. Th2 cytokines (IL-4/IL-13) play the most fundamental role in this process. They bind to corresponding receptors on the surface of B cells, activating the JAK/(signal transducer and activator of transcription) STAT signaling pathway. This initiates the crucial Ig class switching and recombination, causing B cells to transition from producing IgM, IgG and other types to specifically generating IgE (7). Furthermore, focal infection as an initiating factor amplifies Th2 inflammation and indirectly promotes the dysregulation of IgE-mediated immune responses by releasing superantigens to activate T cells and inducing Treg dysfunction. Post-infection resolution of Th2 markers correlates with symptom improvement (8). Results from a cohort study involving 24,997 cases exposed to biological agents indicate that patients receiving IL-17 inhibitors and TNF-α inhibitors face the highest risk of developing paradoxical psoriasis (9). Infliximab may induce immune complex-mediated Type III hypersensitivity reactions and shift the Th1/Th2 immune balance (10), whilst also potentially impairing neutrophil function. This may lead to the reactivation of slow-growing microorganisms such as Propionibacterium acnes, exacerbating cutaneous manifestations (11). Secukinumab may disrupt cytokine homeostasis by inhibiting IL-17A’s immune surveillance function, potentially increasing upstream cytokines such as IL-23 and Interferon(IFN)-α (12), thereby exacerbating Th2 immunological skewing and elevated IgE levels (13). Within the local microenvironment, Th17 cells undergo a phenotypic shift towards Th2, while IL-4 attenuates IL-23/Th17 axis activity by inhibiting STAT3 phosphorylation (14). This mechanism explains the limited efficacy observed with IL-17/IL-23 inhibitors.

A substantial amount of clinical literature supports the use of JAK/STAT inhibitors for treating SAPHO syndrome. These agents effectively alleviate clinical symptoms, reduce inflammatory marker levels, and demonstrate good overall tolerability in patients, as summarised in **Table 3**. The therapeutic rationale for employing upadacitinib, a highly selective JAK1 inhibitor, in SAPHO syndrome lies in its broad inhibitory effects on multiple inflammatory signaling pathways. The drug competitively inhibits the binding of adenosine triphosphate(ATP) to the tyrosine kinase domain of JAK1 (15). This high selectivity enables it to effectively block the signaling of multiple pro-inflammatory cytokines while minimizing interference with JAK2/JAK3-dependent physiological processes (16), thereby demonstrating superior therapeutic efficacy and safety. Upadacitinib addresses paradoxical reactions induced by biologics by concurrently inhibiting the signal transduction of multiple Th2 cytokines—including IL-4, IL-13, thymic stromal lymphopoietin(TSLP), IL-5, and IL-31—thereby suppressing their biological effects (17). This dual action reverses both upstream Th2 polarization and downstream inflammatory responses, ultimately ameliorating Th2 inflammation and cytokine dysregulation (18).

**Table 3:** Literature summary of JAK/STAT inhibitor therapy for SAPHO syndrome.

Pt	Sex	Age(yrs)	Biological agent	Previously administered medications	Skin symptoms	Inflammatory markers	Onset time and dosage of biological agents(weeks)	Time to remission(weeks)	concomitant diseases
1~8 (33)	/	/	upadacitinib	/	Significant reduction in PPPASI score	/	4weeks, 15mg QD	12weeks	/
9~14 (34)	F	means 50.8	upadacitinib	Adalimumab, Secukinumab, Infliximab, etc.	skin lesions resolved or subsided	reduce	4weeks, 15~30mg QD	24weeks	/
15 (35)	F	27	upadacitinib	Etoricoxib, Alendronate	skin lesion symptoms disappeared	/	12weeks, 15mg QD	24weeks	/
16 (36)	F	44	tofacitinib	etanercept, MTX	Significant improvement	reduce	4weeks, 5mg BID	12weeks	/
17 (37)	F	62	tofacitinib	bisphosphonates, MTX,TwHF	PPP healed	reduce	4weeks, 5mg BID	24weeks	/
18~29 (38)	F	means 39.4	tofacitinib	Infliximab, NSAIDs, MTX	skin lesions alleviated	reduce	5mg BID	13.6weeks	/
30~42 (39)	F	means 39.7	tofacitinib	/	Significant reduction in PPPASI score	reduce	5mg BID	12weeks	/
43 (40)	F	29	tofacitinib	NSAIDs, glucocorticoids, IL-6 inhibitors	Significant improvement	reduce	3weeks, 5mg BID	16weeks	LAM
44 (41)	M	36	tofacitinib	MTX, adalimumab	rash subsided significantly	reduce	4weeks, 5mg BID	/	AS
45 (42)	M	36	tofacitinib	MTX	skin symptoms relieved	reduce	8weeks, 5mg BID	24weeks	Takayasu arteritis
**46 (**43)	M	28	tofacitinib	corticosteroids,NSAIDs	acne alleviated	/	5mg BID	80weeks	relapsing polychondritis
47 (44)	F	25	tofacitinib	methylprednisolone	ulcer was healed,skin lesions were subsided	reduce	8weeks, 5mg BID	/	Henoch–Schönlein Purpura
48 (30)	F	31	tofacitinib	Yisaipu, secukinumab	PPP were eradicated	reduce	2weeks,5mg BID	24weeks	AS
49 (45)	F	41	Baricitinib	/	/	/	2mg QD	52weeks	/
50 (46)	F	32	Baricitinib	acitretin capsules, adalimumab	skin lesions disappeared	reduce	2weeks, 2mg BID	12weeks	/
51~55 (47)	/	means 43.2	Baricitinib	NSAIDs, TNFi, bisphosphonate, MTX,ect.	cutaneous manifestation improved	reduce	2mg QD	12weeks	/

MTX, methotrexate; TwHF, tripterygium wilfordii hook F; NSAIDs, non-steroidal anti-inflammatory drugs; IL, interleukin; TNFi, tumor necrosis factor inhibitor; PPPASI, palmoplantar pustulosis area and severity index; QD, quaque die; BID, bis in die; AS, ankylosing spondylitis; LAM, lymphangioleiomyomatosis.

In summary, the emergence of paradoxical reactions accompanied by Hyperimmunoglobulinemia E in SAPHO syndrome patients during biologic therapy reflects a complex immunological dysregulation. We posit that this phenomenon may be associated with the following mechanisms: first, a pre-existing Th2 inflammatory predisposition in patients, characterized by overactivation of the Th2 immune pathway (IL-4/IL-13), which directly drives antibody class switching in B cells; second, the administration of biologics may disrupt immune homeostasis, leading to impaired immune surveillance and further exacerbating Th2 bias and cytokine imbalance; additionally, focal infections as initiating factors amplify Th2 inflammation. Any therapeutic intervention that disrupts immune equilibrium risks being “counteracted” or even “reversed” by the Th2 pathway, triggering paradoxical manifestations and hypersensitivity reactions.

This case report presents the first documented instance of a patient with SAPHO syndrome developing a severe paradoxical cutaneous reaction accompanied by hyperimmunoglobulinaemia following treatment with IL-17 and TNF-α inhibitors, ultimately controlled by the JAK1 inhibitor upadacitinib. This unique clinical trajectory not only offers novel therapeutic insights for such multiply refractory cases but also suggests a potential biologically triggered subtype centred on Th2 immunological bias.

However, this case study carries limitations. Firstly, as a single case report, it is inherently observational and descriptive, incapable of establishing causality and thus offering limited generalisability. The success of this case carries selection bias, and the influence of placebo effects, natural disease fluctuations, or unidentified confounding factors cannot be entirely excluded. Secondly, our interpretation of the Th2-biased mechanism relies primarily on serum IgE as an indirect biomarker, lacking direct immunological evidence from lesion tissue. Finally, despite a six-month follow-up period, this remains insufficient for assessing long-term efficacy and safety in a chronic condition.

Moreover, when considering the use of upadacitinib or similar JAK inhibitors for SAPHO syndrome, their known class-related risks must be systematically assessed and managed. Based on large clinical trial data from diseases such as rheumatoid arthritis, risks requiring attention with JAK inhibitors include: increased risk of infections, major adverse cardiovascular events and venous thromboembolism, as well as laboratory parameter changes such as dyslipidaemia (19). Therefore, a comprehensive baseline risk assessment must be conducted before clinical use, with close monitoring throughout treatment.

## Conclusion

Conclusively, this case offers significant insights for SAPHO syndrome patients who develop paradoxical reactions to biologic therapy. However, its widespread application requires further validation through additional clinical cases and more extensive research. Treatment decisions should be individualized based on disease characteristics, treatment history, and risk assessment.

## Data Availability

The original contributions presented in the study are included in the article/supplementary material. Further inquiries can be directed to the corresponding authors.
